# Novel PET imaging biomarkers as predictors of postoperative recurrence in lung adenocarcinoma

**DOI:** 10.1186/s12885-025-14263-0

**Published:** 2025-05-14

**Authors:** Cheng Zheng, Jiangfeng Miao, LiuWei Xu, Yujie Cai, BingShu Zheng, ZhongHua Tan, ChunFeng Sun

**Affiliations:** Department of Nuclear Medicine, Affiliated Hospital of Nantong University, ChongChuan District, No. 20 of Xisi Road, ChongChuan District, Nantong City, Jiangsu 226001 China

**Keywords:** Lung adenocarcinoma, ^18^F-FDG PET/CT, Prognosis

## Abstract

**Background:**

The exploration of biomarkers is of crucial importance for the prognosis of cancer patients. The objective of this study was to ascertain the predictive value of positron emission tomography (PET) image-derived biomarkers, specifically the normalized distances from the hot spot of radiotracer uptake to the tumor centroid (NHOC) and the tumor perimeter (NHOP), in forecasting the recurrence risk and disease-free survival (DFS) in patients with operable stage IA–IIIA lung adenocarcinoma (LUAD).

**Methods:**

A retrospective analysis was conducted on 164 patients with surgically treated pathologically confirmed stage IA–IIIA LUAD, all of whom had prior ^18^F-Fluorodeoxyglucose Positron Emission Tomography/Computed Tomography (^18^F-FDG PET/CT) scans. In addition to conventional PET/CT parameters, we assessed the normalized distances from the maximum SUV to both the tumor centroid (NHOCmax) and the tumor perimeter (NHOPmax) as observed in the PET/CT images.

**Results:**

A total of 164 patients were included, with a median age of 65 years. NHOPmax exhibited the highest AUC of 0.682 (95% CI: 0.578–0.785), with a sensitivity of 78.8%. Correlation analysis showed that NHOPmax had low correlations with other metabolic parameters such as SUVmax, TLG, and MTV. In both univariate and multivariate analyses, NHOPmax was significantly associated with postoperative outcomes (*P* < 0.001, odds ratio 0.033). Survival analysis indicated that NHOPmax was an independent predictor of DFS (HR = 0.399, *P* < 0.05), with higher NHOPmax (> 0.43) associated with significantly better survival (*P* < 0.0001).

**Conclusion:**

NHOPmax quantified from ^18^F-FDG PET/CT scans, could be a promising predictor of postoperative recurrence in patients with resectable LUAD.

## Introduction

The Cancer Statistics report for 2024 indicates that lung cancer continues to be the primary trigger of deaths associated with cancer, with lung adenocarcinoma (LUAD) recognized as the most commonly found histological subtype [1]. Despite undergoing curative surgery, local or distant recurrence occurs in 20%−45% of patients with stage IA–IIIA lung cancer [2, 3]. For individuals diagnosed with Non-small cell lung carcinoma (NSCLC), accurate staging plays a pivotal role. It not only steers the choice of surgical approach and adjunct therapies but also facilitates the assessment of patient outcomes. At present, the TNM staging system, regarded as the most effective method for tumor staging globally, is frequently employed to evaluate the prognosis of malignant tumors. For patients with LUAD, identifying prognostic factors is vital, as the TNM system alone fails to account for the differences in survival rates among lung cancer patients [4]*.*

Current guidelines recommend the use of ^18^F-Fluorodeoxyglucose Positron Emission Tomography/Computed Tomography (^18^F-FDG PET/CT) for initial staging in all patients with NSCLC [5–7]. Earlier research has validated the significance of routine PET parameters in forecasting lung cancer relapse and evaluating prognostic risks, such as standardized uptake value (SUV), metabolic tumor volume (MTV), and total lesion glycolysis (TLG) [8–11].

A recent investigation created a mathematical model of tumor growth, revealing that as cancer progresses, the highest metabolic activity shifts toward the outer edge of the tumor [12]. Consequently, as a malignant tumor develops over time, the normalized distance from the radiotracer uptake hot spot to the tumor centroid (NHOC) increases, whereas the normalized distance from the hot spot to the tumor perimeter (NHOP) decreases. However, the prognostic importance of NHOC and NHOP parameters in forecasting Postoperative Recurrence Risk in LUAD remains unreported to date.

The aim of this study was to appraise the clinical relevance of NHOCmax and NHOPmax, which were derived from pretreatment ^18^F-FDG PET/CT scans, in forecasting the postoperative recurrence risk of resectable LUAD. We present this article in accordance with the TRIPOD reporting checklist.

## Methods

### Patients

A retrospective analysis was carried out involving 621 patients with LUAD who had undergone ^18^F-FDG PET/CT scans at the Department of Nuclear Medicine in our hospital, with their diagnoses confirmed pathologically from July 2016 to October 2021.The retrospective study was endorsed by the Ethics Committees of our centers, which also waived the requirement for obtaining informed consent.

The inclusion criteria were as follows: (1) Baseline ^18^F-FDG PET/CT was performed at our hospital before surgery within 6 weeks before surgery; (2) Surgical resection of lung lesions confirmed as stage IA–IIIA adenocarcinoma at our hospital; (3) Complete clinical and pathological data. The exclusion criteria were as follows: (1) Preoperative neoadjuvant therapy; (2) Loss of follow-up after surgery; (3) history of another malignant disease. Baseline clinical data included age, gender, surgical type, and tumor stage. Dedicated lung pathologists performed an analysis of the resected surgical specimens. The histological classification of NSCLC was carried out based on the criteria established by the World Health Organization (WHO) [13]. The histopathological grading was carried out in accordance with the 8 th edition of the American Joint Committee on Cancer [14]. In this study, disease-free survival (DFS) was adopted as the primary observational indicator, specifically referring to the duration from the day of surgery until the occurrence of a landmark event that clearly indicates treatment failure. The follow-up work was completed by October 2024. A minimum follow-up period of 3 years was required for all included patients. During this period, patients followed a standardized follow-up procedure: in the first two years after surgery, to promptly detect changes in the condition, a comprehensive follow-up assessment was conducted every three months; from the second year to the fifth year, based on the stage characteristics of the condition, follow-up was carried out every six months; after five years, considering that the condition was relatively stable, routine follow-up was performed once a year.

### ^*1*8^F-FDG PET/CT image acquisition

Participants were required to fast for a minimum of 6 h while maintaining their blood glucose levels below 150 mg/dL before receiving an intravenous injection of ^18^F-FDG at a dose of 4.07 MBq/kg. PET/CT imaging was performed about 60 min post-injection, covering the area from the base of the skull to the upper thigh, using a GE Healthcare Discovery™ 710 64-slice spiral CT scanner.

The CT configuration included a slice thickness of 3.75 mm and utilized a matrix dimension of 512 × 512. For the PET imaging, six to eight-bed positions were arranged according to the height of the patient, with each scan taking between 2 and 3 min and employing a matrix size of 192 × 192.

### Data preprocessing

A retrospective analysis of ^18^F-FDG PET/CT images was conducted by two seasoned physicians specialized in nuclear medicine, utilizing the open-source LIFEx software version 7.6.9 (www.lifexsoft.org), which adheres to the Image Biomarker Standardisation Initiative guidelines [15].

**Fig. 1:**
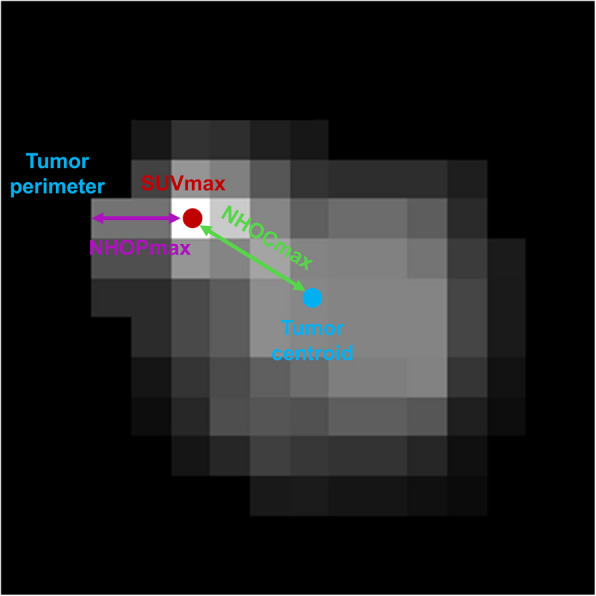
Diagrams depicting the meanings of NHOCmax and NHOPmax

Before conducting the image analysis, the PET images were reconstructed to a voxel dimension of 3 × 3 × 3 mm. The initial segmentation was performed by a nuclear medicine physician with 8 years of experience, and all contours were subsequently reviewed and independently verified by a senior nuclear medicine expert with over 20 years of clinical experience to ensure consistency and accuracy. From the primary cancer lesion that was metabolically active, three conventional PET parameters (SUVmax, MTV, and TLG) were computed. As shown in Fig. 1, the normalized distance from the maximum standardized uptake value to the tumor centroid (NHOCmax) refers to the distance from the voxel containing SUVmax to the tumor centroid, calculated by dividing this distance by the radius of a theoretical sphere that has an equivalent volume to the tumor. Conversely, the normalized distance from the maximum standardized uptake value to the tumor perimeter (NHOPmax) represents the minimum Euclidean distance distance from the SUVmax voxel to the tumor perimeter, also divided by the radius of the same hypothetical sphere. The volume of the lesion that was metabolically active was automatically delineated using a threshold established at 40% of SUVmax, and the tumor boundary was defined as the outermost layer of connected voxels within this segmentation.

### Statistical analysis

Quantitative variables are represented as mean ± standard deviation (− X ± SD) or median (quartile) [M (Q1, Q3)], while qualitative variables are expressed as frequencies (percentages). The relationships between NHOCmax and NHOPmax with other features were examined through Spearman correlation coefficients (r). Univariate and multivariate logistic regression analyses were utilized to identify independent predictors of recurrence risk. In order to assess how effective ^18^F-FDG PET/CT parameters were at predicting recurrence risk, a dedicated evaluation method was applied. More precisely, the area under the receiver operating characteristic (ROC) curve, known as AUC, was calculated. At the same time, the ideal cut-off value identified through the Youden index was employed. The prognostic relevance of PET/CT parameters for predicting DFS was evaluated through univariate and multivariate Cox proportional hazards regression analyses. For predicting recurrence risk, the cut-off value established by ROC curve analysis was used in the Kaplan–Meier analysis to estimate the DFS curves. Comparisons of DFS curves between groups were conducted with the log-rank test. All statistical analyses were deemed significant at *p* < 0.05. Analyses were conducted using Python (version 3.7).

## Results

### Patient characteristics

The distribution of PET parameters and characteristics of the enrolled patients are summarized in Table 1. A total of 164 patients participated in the study. The median age was 65 years (range, 36–85 years). The cohort included 72 males (43.90%) and 92 females (56.10%). TNM staging revealed that stage IA was the most common (51.22%, 84 patients), followed by stage IB (26.22%, 43 patients). T stage distribution showed that 56.71% (93 patients) were classified as T1. N stage distribution indicated that 85.98% (141 patients) were N0. Surgical interventions included lobectomy in 70.73% (116 patients), segmentectomy in 10.37% (16 patients), and sublobar resection in 18.90% (25 patients). The median clinical follow-up duration was 57 months (range, 14–96 months), during which 33 patients (20.1%) experienced clinical events.

**Table 1:** Patient characteristics and PET parameters distribution

**Characteristics**		**164 patients**
**Median age(range)**		65(36–85 years)
**Sex**	**Male**	72(43.90%)
	**Female**	92(56.10%)
**Stage**	**IA**	84(51.22%)
	**IB**	43(26.22%)
	**IIA**	10(6.10%)
	**IIB**	13(7.93%)
	**IIIA**	14(8.54%)
**T Stage**	**T1**	93(56.71%)
	**T2**	55(33.54%)
	**T3**	7(4.27%)
	**T4**	9(5.49%)
**N Stage**	**N0**	141(85.98%)
	**N1**	14(8.54%)
	**N2**	9(5.49%)
**Operation**	**Lobectomy**	116(70.73%)
	**Segmentectomy**	16(10.37%)
	**Sublobar resection**	25(18.90%)
**Adjuvant Therapy**	**No**	143(87.20%)
	**Chemotherapy**	16(9.76%)
	**Targeted Therapy**	5(3.05%)
**CEA**		11.59 ± 30.49
**SUVmax**		6.54 ± 4.62
**TLG**		25.68 ± 76.45
**MTV**		5.43 ± 10.61
**NHOCmax**		0.55 ± 0.28
**NHOPmax**		0.34 ± 0.23

*AUC* area under the receiver operating characteristic curve, *CI* confidence interval

### Correlation analysis of conventional and new PET parameters

**Fig. 2:**
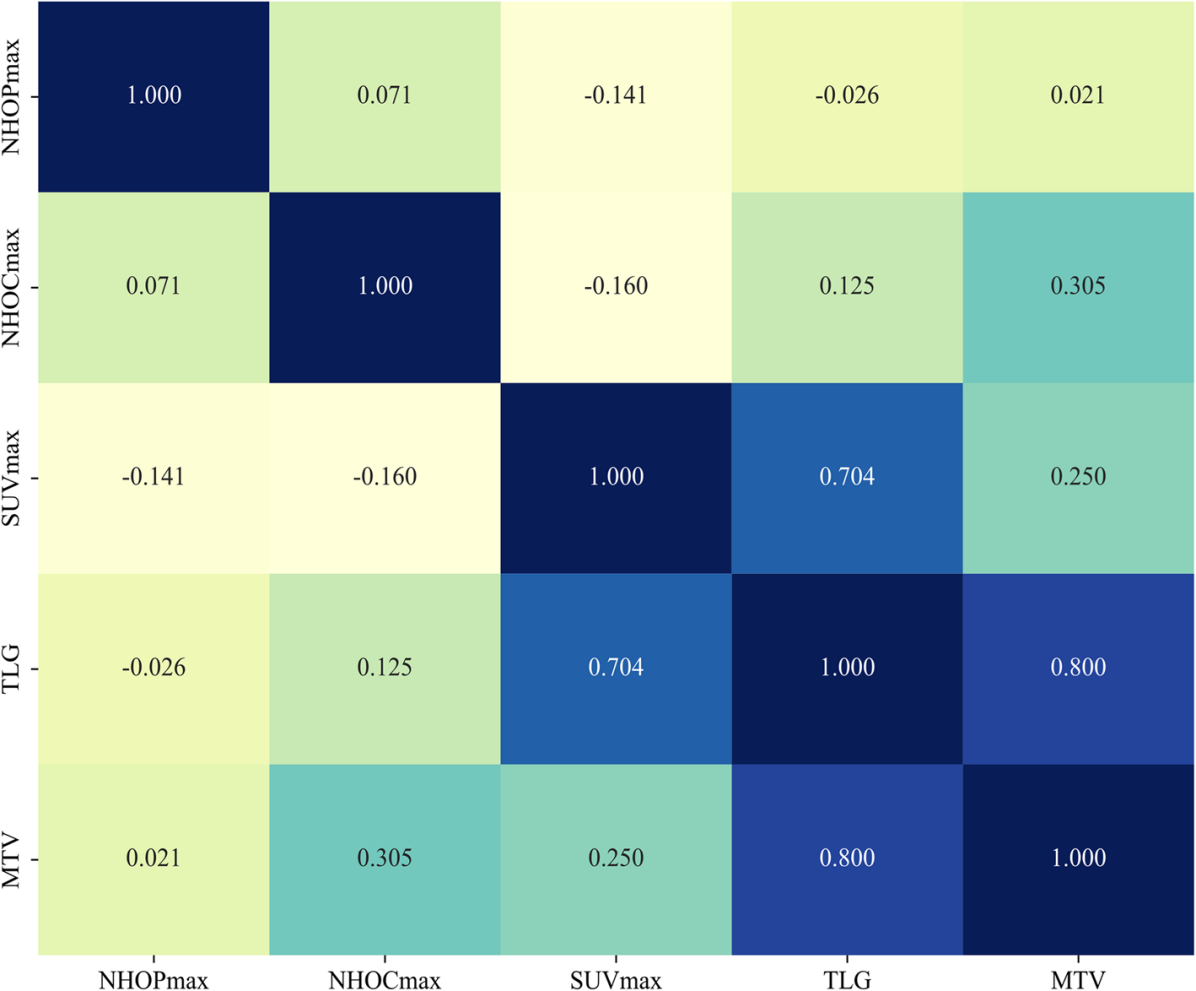
Correlogram of novel and conventional metabolic parameters

Based on the data from Fig. 2, we analyzed the correlations among NHOPmax, NHOCmax, SUVmax, TLG, and MTV. ​NHOPmax​​ was negatively correlated with SUVmax (*r *= −0.141), weakly negatively correlated with TLG (*r* = −0.026), and showed a near-zero correlation with MTV (*r* = 0.021), but none of these correlations reached statistical significance (*p* > 0.05). ​​In contrast, NHOCmax​​ displayed a weak positive correlation with TLG (*r* = 0.125, *p* < 0.05) and a moderate positive correlation with MTV (*r *= 0.305, *p* < 0.05).

### Predictive performance of conventional and new PET parameters

**Fig. 3:**
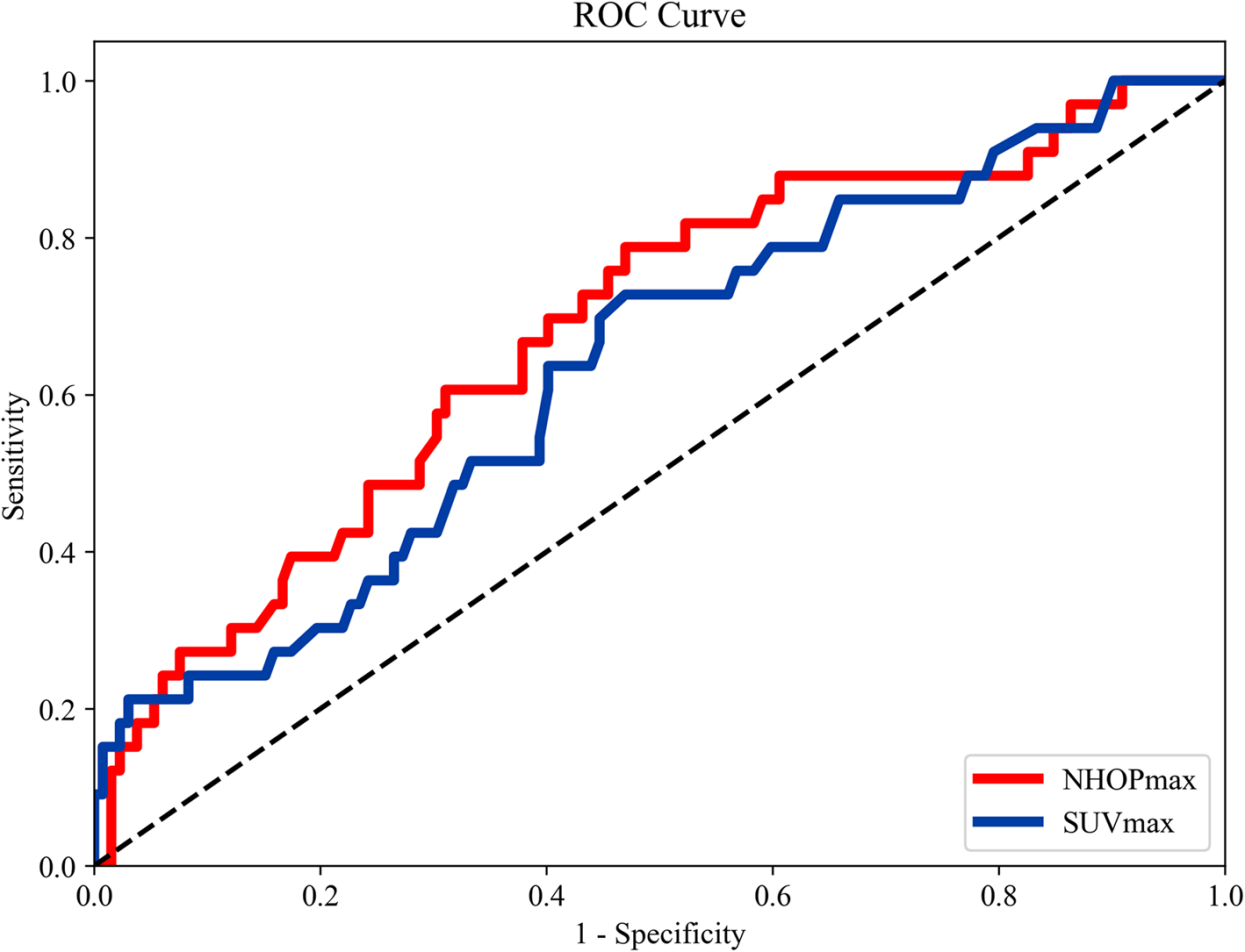
ROC curve assessment of NHOPmax and SUVmax in predicting the risk of recurrence

Analysis of diagnostic parameters revealed that NHOPmax had the highest AUC at 0.682 (95% CI: 0.578–0.785), followed by SUVmax with an AUC of 0.636 (95% CI: 0.522–0.740). TLG and MTV exhibited similar AUC values of 0.557 (95% CI: 0.437–0.670) and 0.554 (95% CI: 0.435–0.665), respectively, while NHOCmax presented an AUC of 0.499 (95% CI: 0.387–0.616), as illustrated in Fig. 3. In terms of specificity, TLG demonstrated the highest value at 85.6%, followed by NHOCmax (62.9%), MTV (59.1%), and both NHOPmax and SUVmax (53.0%). Sensitivity was highest for NHOPmax (78.8%), with SUVmax (72.7%), MTV (54.5%), NHOCmax (48.5%), and TLG (33.3%) showing lower values. A detailed summary of these findings is provided in Table 2.

**Table 2:** Prediction performance of Novel and Conventional Metabolic Parameters

Parameter	AUC (95% Cl)	Sensitivity	Specificity	Cut-off value
NHOPmax	0.682(0.578–0.785)	0.788	0.530	0.430
NHOCmax	0.499(0.387–0.616)	0.485	0.629	0.609
SUVmax	0.636(0.522–0.740)	0.727	0.530	5.800
MTV	0.554(0.435–0.665)	0.545	0.591	3.050
TLG	0.557(0.437–0.670)	0.333	0.856	30.000

*AUC* area under the receiver operating characteristic curve, *CI* confidence interval

### Findings of PET parameters and clinical data

**Fig. 4:**
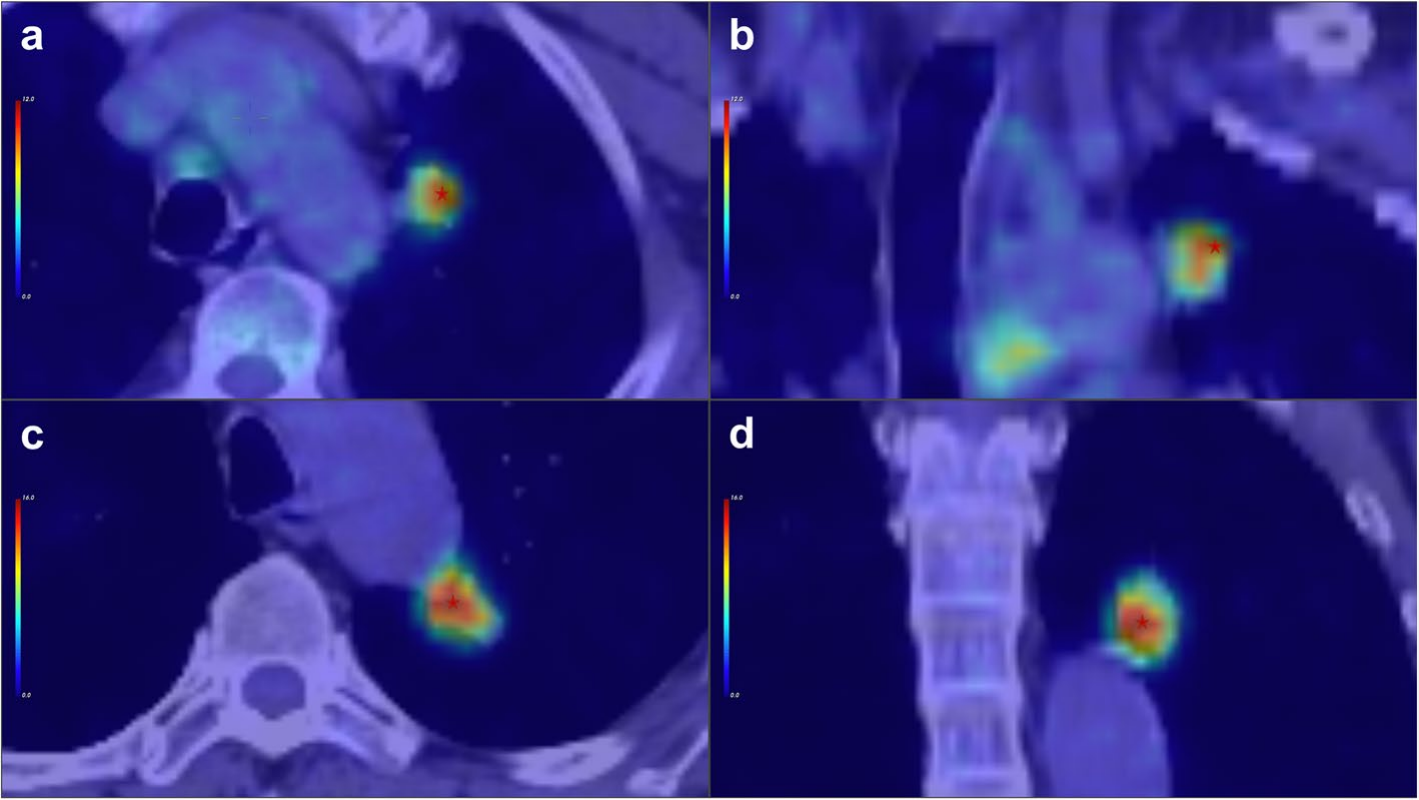
Transaxial (**a**) and coronal (**b**) FDG PET/CT images from a 58-year-old male patient, post-operative pathology confirming IA stage lung adenocarcinoma (LUAD). The patient experienced recurrence 16 months after surgery, with a SUVmax value of 9.50, MTV value of 2.02, TLG value of 11.90, NHOP value of 0.43, and NHOC value of 0.121. Transaxial (**c**) and coronal (**d**) FDG PET/CT images of a 70-year-old male patient, post-operative pathology confirming stage IIA lung adenocarcinoma (LUAD). The patient has been followed for 74 months post-surgery without recurrence, with a SUVmax value of 16.30, MTV value of 3.05, TLG value of 30.00, NHOP value of 0.32, and NHOC value of 0.13. The position of the hotspot for FDG uptake is notably shifted towards the margin in image (**b**). Position of hot spot of FDG uptake is indicated by a red star

NHOPmax was significantly associated with postoperative outcomes in both univariate (*P *< 0.001, odds ratio 0.033, 95% CI: 0.015–0.075) and multivariate analyses (P = 0.002, odds ratio 0.035, 95% CI: 0.006–0.210). Similarly, SUVmax demonstrated significance in both analytical contexts. In contrast, NHOCmax showed relevance only in the univariate analysis (P < 0.001, odds ratio 0.112, 95% CI: 0.063–0.200). Additionally, T stage, surgical approach, TNM stage, and metabolic tumor volume (MTV) were significant only in the univariate analysis. The independent predictors of postoperative recurrence, as summarized in Table 3, include NHOPmax and SUVmax. Figure 4 provides PET/CT scans illustrating tumors with varying levels of NHOCmax and NHOPmax.

**Table 3:** Univariate and multivariate logistic regression analyses of PET parameters and clinical data

Parameter	Univariate analysis		Multivariate analysis	
	*P*-value	Odds ratio (95% CI)	*P*-value	Odds ratio (95% CI)
**NHOPmax**	< 0.001	0.033 (0.015–0.075)	0.002	0.035 (0.006–0.210)
**NHOCmax**	< 0.001	0.112 (0.063–0.200)	0.951	0.958 (0.308–2.989)
**T Stage**	< 0.001	0.461 (0.374–0.567)	0.109	0.496 (0.242–1.018)
**Operation**	< 0.001	0.446 (0.357–0.557)	0.759	1.074 (0.733–1.573)
**Adjuvant Therapy**	0.192	0.628(0.349–1.130)		
**Stage**	< 0.001	0.623 (0.506–0.767)	0.528	1.163 (0.785–1.723)
**MTV**	0.028	0.948 (0.910–0.987)	0.153	1.030 (0.996–1.065)
**SUVmax**	< 0.001	0.894 (0.859–0.930)	0.036	1.100 (1.020–1.186)
**TLG**	0.648	0.999(0.996–1.002)		
**N Stage**	0.079	0.578 (0.346–0.967)		
**CEA**	0.851	0.999(0.991–1.007)		

*CI* confidence interval

### Survival analysis

**Fig. 5:**
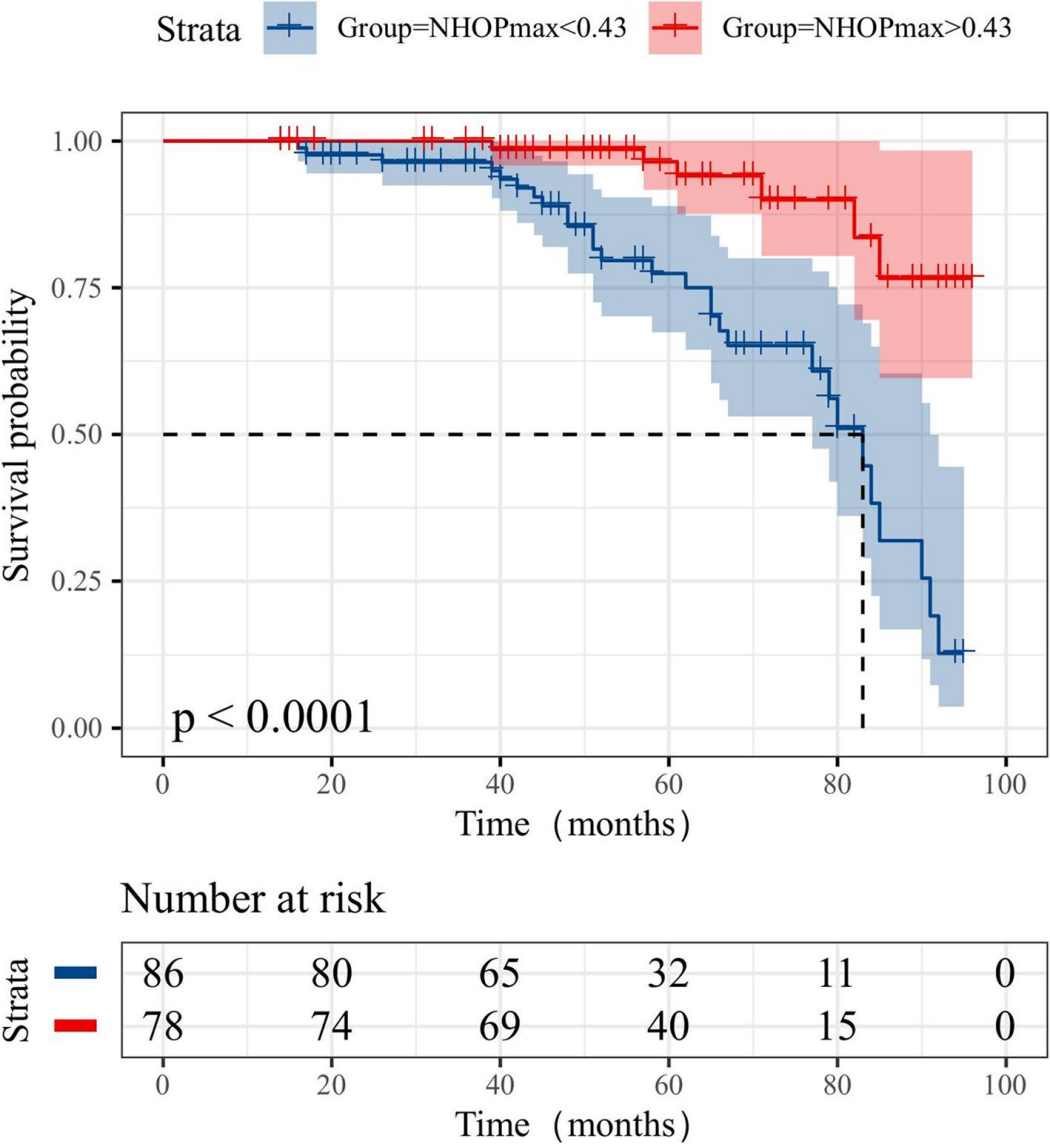
Kaplan–Meier curves of DFS according to NHOPmax

The results of the univariate survival analysis indicated that NHOPmax demonstrated a significant protective effect against lung cancer recurrence, with an HR of 0.079 (95% CI: 0.017–0.360; *P* < 0.05). Additionally, N stage and SUVmax were significantly associated with recurrence in the univariate analysis (*P* < 0.05). However, TNM stage did not reach statistical significance (*P* = 0.073). In the multivariate survival analysis, N stage remained significantly associated with recurrence, with an HR of 1.423 (95% CI: 1.018–1.988; *P* < 0.05). Similarly, NHOPmax continued to show a protective effect, with an HR of 0.399 (95% CI: 0.162–0.979; *P* < 0.05). SUVmax, which was significant in the univariate analysis, did not retain its significance in the multivariate model (HR = 1.033, 95% CI: 0.990–1.077; *P* = 0.131). Other variables, including T stage, TLG, MTV, operation type, adjuvant therapy, and CEA, were not significantly associated with recurrence in either univariate or multivariate analyses. In this analysis, NHOPmax and N stage emerged as statistically significant (P < 0.05), identifying it as an independent predictor of disease-free survival (DFS) (Table 4). Kaplan–Meier survival analysis was performed for two groups based on NHOPmax values (NHOPmax < 0.43 and NHOPmax > 0.43). The log-rank test yielded a P-value below 0.0001, indicating a significant difference in survival probabilities between the two groups, with the NHOPmax > 0.43 group displaying a higher survival probability throughout the observation period (Fig. 5).

**Table 4:** Univariate and multivariate survival analyses for DFS

Parameter	Univariate analysis		Multivariate analysis	
	*P*-value	Hazard ratio (95% CI)	*P*-value	Hazard ratio (95% CI)
**Stage**	0.073	1.204 (0.983–1.475)		
**T Stage**	0.734	1.060 (0.757–1.485)		
**N Stage**	< 0.05	2.162 (1.426–3.279)	< 0.05	1.423 (1.018–1.988)
**SUVmax**	< 0.05	1.082 (1.022–1.144)	0.131	1.033 (0.990–1.077)
**TLG**	0.473	1.001 (0.998–1.004)		
**MTV**	0.991	1.000 (0.974–1.026)		
**Operation**	0.184	1.256 (0.898–1.756)		
**Adjuvant Therapy**	0.521	0.792 (0.388–1.615)		
**CEA**	0.281	1.008 (0.994–1.022)		
**NHOCmax**	0.170	0.486 (0.173–1.364)		
**NHOPmax**	< 0.05	0.079 (0.017–0.360)	< 0.05	0.399 (0.162–0.979)

*CI* confidence interval

## Discussion

In the era of precision medicine, LUAD has emerged as a distinct subtype within the spectrum of lung cancers, demanding specialized attention [16]. It represents a significant proportion of lung cancer cases and is characterized by its own unique biological behavior and clinical course [17]. The prognosis of LUAD patients varies widely and is influenced by multiple factors such as tumor stage and histological grade [18, 19]. Alarmingly, the mortality rate associated with this disease remains high, highlighting the urgent need for more effective prognostic tools and treatment strategies [1].

PET imaging has long been utilized in the management of LUAD, with a plethora of metabolic parameters being investigated for their prognostic value. SUV, MTV, and TLG are among the commonly studied parameters [20, 21]. However, their application has been fraught with challenges. SUV, for instance, is highly susceptible to the influences of tumor heterogeneity and partial volume effects, resulting in inconsistent and unreliable results when used to predict recurrence risk [22]. MTV and TLG also face significant hurdles in accurately reflecting the true biological behavior of tumors due to difficulties in precise tumor delineation and the inherent variability of FDG uptake [23]. In contrast, NHOC and NHOP offer a novel perspective based on the geometric characteristics of tumors, potentially providing more robust and reliable information [12].

Novel biomarkers, NHOC and NHOP, have come to the forefront in the search for improved prognostic capabilities [24, 25]. These biomarkers are inherently linked to the dynamic processes of tumor growth. As LUAD progresses, the distribution of metabolic activity within the tumor undergoes significant changes. The area of highest metabolic activity, typically denoted by SUVmax and SUVpeak, tends to shift towards the tumor edge. This migration can be attributed to the complex interplay of factors within the tumor microenvironment and the process of biological evolution. Increased cellular density and proliferation rates in the central region often lead to hypoxia and nutrient depletion, compelling the more aggressive tumoral clonal populations to migrate towards the periphery in pursuit of more favorable conditions [12]. NHOC and NHOP precisely capture this dynamic alteration by quantifying the distances from these hotspots to the geometric center and the periphery of the tumor respectively, after normalizing for tumor size. In this study, we embark on an in-depth exploration of the potential of NHOCmax and NHOPmax in predicting the postoperative recurrence risk of LUAD.

In this study, we highlight the potential value of NHOPmax as a novel PET/CT-derived biomarker for predicting recurrence in patients with LUAD after surgery. With an AUC of 0.682 and a sensitivity of 78.8%, NHOPmax demonstrates meaningful discriminatory ability and shows improved performance in recurrence prediction compared to traditional PET/CT parameters such as SUVmax, MTV, and TLG. Notably, previous studies have reported conflicting prognostic value for these conventional metrics, largely due to tumor heterogeneity and variability in tumor delineation methods [26, 27]. In contrast, NHOPmax offers a new approach to assessing recurrence risk by capturing distinct tumor characteristics. It is associated with a significantly reduced risk of recurrence (HR = 0.399), and Kaplan–Meier survival analysis shows that patients with NHOPmax values above 0.43 have better disease-free survival. Both univariate (*P* < 0.001) and multivariate (P = 0.002) analyses support its role as an independent prognostic factor. Moreover, NHOPmax shows only weak correlations (correlation coefficients < 0.50) with SUVmax, MTV, and TLG, suggesting that it provides complementary information and additional clinical insight beyond conventional PET/CT metrics.

Importantly, NHOPmax may also offer new perspectives for cancer treatment beyond recurrence risk prediction. Unlike traditional metabolic parameters, higher NHOPmax values are associated with tumor hypoxia, a microenvironmental condition that promotes the accumulation of lactate and ketone bodies—metabolites known to suppress T-cell function. This immunosuppressive state may compromise the efficacy of immune checkpoint inhibitors, indicating that patients with decreased NHOPmax could potentially benefit from therapeutic strategies that combine immunotherapy with metabolic modulation. Thus, NHOPmax not only serves as a prognostic imaging biomarker but also provides mechanistic insights into tumor biology and therapeutic vulnerabilities in LUAD.

Jiménez-Sánchez et al. [28]. demonstrated that the normalized SUVmax to perimeter distance (nSPD) is a prognostic factor for the survival of patients with NSCLC. Undergoing the modification, in our study, NHOPmax serves as a three-dimensional surrogate for nSPD. Notably, NHOPmax exhibits a closer alignment with the actual tumor characteristics. It takes into account the complex three-dimensional architecture of the tumor, providing a more comprehensive representation compared to traditional metrics. By integrating spatial information, NHOPmax can more accurately capture the heterogeneity within the tumor mass. This means it not only reflects the metabolic activity, as indicated by SUVmax, but also factors in the tumor's physical extent and irregularities in its boundary. Consequently, NHOPmax holds the potential to offer enhanced prognostic value, allowing for more precise predictions of patient survival and more informed clinical decision-making in the management of LUAD. Hovhannisyan-Baghdasarian et al. [25]. focused on evaluating NHOP and NHOC features derived from baseline ^18^F-FDG PET/CT in advanced NSCLC patients, demonstrating their robustness to imaging variations and their prognostic value for overall survival, particularly under immunotherapy and targeted therapy. In comparison, our study highlights the predictive value of NHOPmax for postoperative recurrence in patients with resectable LUAD, showing superior performance over conventional PET/CT metrics and confirming its role as an independent prognostic factor. Importantly, while their work addressed late-stage, inoperable disease, our findings extend the application of NHOP to early-stage, surgically treated LUAD. Together, these studies provide complementary evidence supporting NHOP as a robust and clinically meaningful biomarker across the full spectrum of LUAD.

Nevertheless, our study has several limitations. The retrospective design may introduce selection biases. Additionally, SUVpeak, and consequently NHOCpeak and NHOPpeak, could not be calculated for volumes of interest (VOI) with a diameter < 12 mm; thus, these parameters were excluded from our study. Although all VOIs were reviewed by a senior nuclear medicine physician, no formal inter-observer validation was performed, which may affect the reproducibility of the results. Furthermore, although NHOPmax values were normalized using a theoretical sphere of equal volume, tumor morphology, such as spiculated or irregular margins, could still influence these measurements.

## Conclusions

Our study has found that an increase in NHOPmax is associated with a lower risk of postoperative recurrence and a higher DFS in LUAD. This indicates that NHOPmax has potential value in predicting prognosis and may contribute to the development of more targeted treatment strategies for LUAD patients.

## Data Availability

The datasets used and/or analysed during the current study are available from the corresponding author on reasonable request.
